# Data Resource Profile: World Health Organization Health Inequality Data Repository

**DOI:** 10.1093/ije/dyad078

**Published:** 2023-06-15

**Authors:** Katherine Kirkby, Nicole Bergen, Andreia Baptista, Anne Schlotheuber, Ahmad Reza Hosseinpoor

**Affiliations:** Department of Data and Analytics, Division of Data, Analytics and Delivery for Impact, World Health Organization, Geneva, Switzerland; Department of Data and Analytics, Division of Data, Analytics and Delivery for Impact, World Health Organization, Geneva, Switzerland; Department of Data and Analytics, Division of Data, Analytics and Delivery for Impact, World Health Organization, Geneva, Switzerland; Department of Data and Analytics, Division of Data, Analytics and Delivery for Impact, World Health Organization, Geneva, Switzerland; Department of Data and Analytics, Division of Data, Analytics and Delivery for Impact, World Health Organization, Geneva, Switzerland

Key Features

- Recently updated and expanded, the World Health Organization (WHO) Health Inequality Data Repository is a large repository for datasets of disaggregated data, covering a diversity of topics, dimensions of inequality and populations.
- As of 2023, the Health Inequality Data Repository covers over 2000 indicators and 22 dimensions of inequality. It includes populations across all world regions and in selected countries.
- Featured topics include: Sustainable Development Goals (SDGs); COVID-19; reproductive, maternal and child health; immunization; HIV, tuberculosis and malaria; adult health; health care; burden of disease; disability; environmental health; WHO Thirteenth General Programme of Work; and other health determinants.
- All datasets are publicly available and are accessible through the Health Equity Assessment Toolkit (HEAT), a software that facilitates interactive data exploration, analysis and reporting.
- Updates to the Health Inequality Data Repository are done about once a year.
- All datasets (and accompanying metadata) are freely accessible for download through the Health Inequality Monitor website [https://www.who.int/data/inequality-monitor/data] as well as via an Open Data Protocol (OData) application programming interface (API).

## Data resource basics

Disaggregated health data are a central requirement for health inequality monitoring, which is the process of quantifying and assessing health inequalities in a defined population. These data, which show how health varies across population subgroups, help with the identification of situations of health inequity, where there are preventable and unfair differences in health, wellbeing, and access to quality health services. Sustainably addressing health inequities is part of major global health and development initiatives, including the United Nations 2030 Agenda for Sustainable Development.

The World Health Organization (WHO) Health Inequality Data Repository was set up to provide access to a large selection of publicly available disaggregated data across various health and health-related topics, encompassing diverse dimensions of inequality. It also facilitates the expanded use of the Health Equity Assessment Toolkit (HEAT) software application for health inequality monitoring.^1^ HEAT enables the assessment of health inequalities, allowing users to explore disaggregated data, calculate summary measures of inequality, perform benchmarking between selected settings and generate and export tables and graphs.^2^

The Health Inequality Data Repository builds upon and replaces the WHO Health Equity Monitor (HEM).^3^ HEM was launched in 2013, containing disaggregated data pertaining to reproductive, maternal, newborn and child health indicators, disaggregated by dimensions of inequality including education, economic status, place of residence, subnational region and child’s sex (where applicable). The data contained within HEM were sourced from multi-country household health surveys and are comparable across countries and over time.

The Health Inequality Data Repository, launched in 2023, is the largest collection of publicly available disaggregated data about health and determinants of health (Table 1).^4^ It expands upon the selection of health topics covered in HEM: in addition to reproductive, maternal, newborn and child health, these include Sustainable Development Goals (SDGs); COVID-19; immunization; HIV, tuberculosis and malaria; adult health; health care; burden of disease; disability; environmental health; WHO Thirteenth General Programme of Work; and other health determinants. In total, the Health Inequality Data Repository contains data from 16 sources, on more than 2000 indicators. Detailed metadata for all indicators featured in the Health Inequality Data Repository are available at: [https://www.who.int/data/inequality-monitor/data]. The Health Inequality Data Repository datasets encompass diverse dimensions of inequality (as per data availability), including age, birth order, city, disability, economic status, education, employment status, employment type, ethnicity/race/caste, health worker status, marital status, migratory status, number of living children, place of residence, religion, sex and subnational region. Double disaggregation—the disaggregation of an indicator by two dimensions of inequality, such as sex and economic status—is also available for some indicators. The populations represented by data in the Health Inequality Data Repository cover all world regions. The date of data collection ranges from 1950 to 2022. All datasets and their accompanying metadata are freely accessible for download through the Health Inequality Monitor website and an Open Data Protocol (OData) application programming interface (API), or can be explored directly through HEAT.^4^ Annual updates are planned, noting that several data sources are updated on an annual basis.

**Table 1.** Characteristics of datasets featured in the World Health Organization Health Inequality Data Repository, as of 2023 launch

Dataset	Original source	Number of indicators^a^	Dimensions of inequality^b^	Number of countries^c^
**SDGs**				

Sustainable Development Goals	United Nations Statistics SDG Indicators Database^d^	95	Age, city, disability, economic status, education, migratory status, place of residence, sex	194

**WHO Global Health Observatory**				

WHO Global Health Observatory	WHO GHO^e^	138	Age, economic status, place of residence, sex	194

**COVID-19**				

COVID-19 burden, behaviours, and testing^f^	UMD-CTIS, in partnership with Facebook^g^	12	Age, education, gender, health worker status, place of residence	109
COVID-19 vaccination^f^	UMD-CTIS, in partnership with Facebook^g^	17	Age, education, gender, health worker status, place of residence	109
COVID-19 related mental health and financial worry^f^	UMD-CTIS, in partnership with Facebook^g^	7	Age, education, gender, health worker status, place of residence	109
COVID-19 case and death rates^h^	WHO COVID-19 Detailed Surveillance Data Dashboard^i^	2	Age, sex	130
COVID-19 case fatality ratios^h^	WHO COVID-19 Detailed Surveillance Data Dashboard^i^	1	Age, sex	111

**Reproductive, maternal, newborn and child health**				

Reproductive, maternal, newborn and child health	Re-analysis of DHS, MICS, and RHS by the WHO Collaborating Center for Health Equity Monitoring (International Center for Equity in Health, Federal University of Pelotas); DHS Program; UNICEF Data Warehouse^j^	69	Age, birth order, economic status, education, place of residence, sex, subnational region	135
Reproductive, maternal, newborn and child health	WHO GHO^e^	7	Age, sex	194
Child malnutrition	UNICEF/WHO/World Bank Joint Malnutrition Estimates^k^	5	Age, economic status, education, place of residence, sex, subnational region	139
Under-five mortality	UN IGME^l^	1	Economic status, sex	194
Subnational under-five mortality – first administrative level	UN IGME^l^	1	Subnational region	22
Subnational under-five mortality - second administrative level	UN IGME^l^	1	Subnational region	17
Subnational anaemia prevalence geospatial estimates	IHME^m^	2	Subnational region	89
Subnational breastfeeding prevalence geospatial estimates	IHME^m^	1	Subnational region	102
Subnational diarrhoea prevalence, incidence and mortality geospatial estimates	IHME^m^	3	Subnational region	109

**Immunization**				

Childhood immunization	Re-analysis of DHS, MICS, and RHS by the WHO Collaborating Center for Health Equity Monitoring (International Center for Equity in Health, Federal University of Pelotas)	18	Age, economic status, education, place of residence, sex, subnational region	110
Subnational Diphtheria–Tetanus–Pertussis immunization dropout rates	Administrative data shared with WHO/UNICEF through the Joint Reporting Form process in 2017 and 2018	1	District quintile	75

**HIV, TB and malaria**				

HIV epidemiological estimates	UNAIDS/UNICEF/WHO estimates^n^	8	Age, sex	147
HIV/AIDS	DHS Program and UNICEF Data Warehouse^j^	19	Age, economic status, education, employment status, marital status, place of residence, sex, subnational region	111
Sub-Saharan Africa subnational HIV incidence and mortality geospatial estimates	IHME^m^	2	Subnational region	44
Tuberculosis	Country reported estimates, DHS, MICS, RHS, TB patient cost surveys, TB prevalence surveys, and WHO estimates	10	Age, economic status, education, place of residence, sex, TB drug resistance status	194
Malaria	DHS Program and UNICEF Data Warehouse^j^	20	Age, economic status, education, place of residence, sex, subnational region	81
Subnational malaria prevalence, incidence and mortality geospatial estimates	IHME^m^	5	Subnational region	104

**Adult health**				

Adult health and nutrition	DHS Program^j^	8	Age, economic status, education, marital status, place of residence, subnational region	76
Alcohol	WHO GHO^e^	23	Sex	192
Tobacco	WHO GHO^e^	23	Sex	193
Noncommunicable diseases and risk factors	WHO GHO^e^	72	Sex	194
Adult health	Eurostat, the statistical office of the European Union^o^	26	Age, economic status, education, place of residence, sex	36
Self-reported health status	OECD^p^	3	Age, economic status, education, sex	41

**Health care**				

Health care quality, resources and expenditure	OECD^p^	67	Age, sex	48
Health care system and access	WHO GHO^e^	20	Age, economic status, place of residence, sex	181
Health care access	DHS Program^j^	10	Age, economic status, education, employment status, marital status, number of living children, place of residence, subnational region	68
Health care	Eurostat, the statistical office of the European Union^o^	17	Age, economic status, education, place of residence, sex	36

**Burden of disease**				

Disease mortality rates, by age	WHO Global Health Estimates^q^	27	Age	183
Disease mortality rates, by sex	WHO Global Health Estimates^q^	154	Sex	183
Disability-adjusted life years, by age	WHO Global Health Estimates^q^	27	Age	183
Disability-adjusted life years, by sex	WHO Global Health Estimates^q^	154	Sex	183
Years of healthy life lost due to disability, by age	WHO Global Health Estimates^q^	27	Age	183
Years of healthy life lost due to disability, by sex	WHO Global Health Estimates^q^	154	Sex	183
Years of life lost from mortality, by age	WHO Global Health Estimates^q^	27	Age	183
Years of life lost from mortality, by sex	WHO Global Health Estimates^q^	154	Sex	183
Life expectancy and mortality	WHO GHO^e^	29	Sex	194
Disease incidence estimates	IHME Global Burden of Disease study^r^	193	Sex	194
Disease prevalence estimates	IHME Global Burden of Disease study^r^	193	Sex	194

**Disability**				

Indicators disaggregated by disability	Eurostat, the statistical office of the European Union^o^	43	Disability	36
Indicators among people with disabilities	Eurostat, the statistical office of the European Union^o^	43	Age, sex	36

**Environmental health**				

Water, sanitation and hygiene (WASH)	WHO/UNICEF JMP^s^	36	Place of residence	176
Environmental health	WHO GHO^e^	26	Place of residence, sex	194

**WHO General Programme of Work**				

WHO Thirteenth General Programme of Work, WHO’s strategy for the period 2019-2025	Re-analysis of DHS, MICS and RHS; WHO Global Health Estimates^q^; WHO GHO^e^; WHO/UNICEF JMP^s^; UNICEF/WHO/World Bank Joint Malnutrition Estimates^k^; UNAIDS/UNICEF/WHO HIV incidence estimates^n^	37	Age, economic status, education, place of residence, sex, subnational region	194

**Beyond the health sector**				

Health determinants	DHS Program and UNICEF Data Warehouse^j^	53	Age, economic status, education, employment type, marital status, number of living children, place of residence, sex, subnational region	194
Health determinants	The World Bank Data Catalog^t^	64	Economic status, place of residence, sex	192
Health determinants	Eurostat, the statistical office of the European Union^o^	24	Age, sex	36
Health determinants	OECD^p^	25	Age, education, sex	41
Child protection	DHS Program and UNICEF Data Warehouse^j^	37	Age, economic status, education, employment status, marital status, number of living children, place of residence, religion, sex, subnational region	190
Women’s empowerment index	Re-analysis of DHS by the WHO Collaborating Center for Health Equity Monitoring (International Center for Equity in Health, Federal University of Pelotas)	9	Age, economic status, education, place of residence, subnational region	64
Development indices (Gini coefficient, Human Development Index, Gender Development Index, Theil-T, and International Wealth Index)	Global Data Lab^u^	10	Economic status, place of residence, poverty status, subnational region	164
Development indicators	Global Data Lab^u^	18	Economic status, place of residence, poverty status, subnational region	164
Multidimensional Poverty Index	UNDP and OPHI^v^	2	Age, ethnicity/race/caste, place of residence, sex of household head, subnational region	110

DHS: Demographic and Health Survey; GHO: Global Health Observatory; IHME: Institute for Health Metrics and Evaluation; JMP: Joint Monitoring Programme for Water Supply, Sanitation and Hygiene; MICS: Multiple Indicator Cluster Surveys; OECD: Organisation for Economic Co-operation and Development; OPHI: Oxford Poverty and Human Development Initiative; RHS: Reproductive Health Surveys; SDG: Sustainable Development Goal; TB: tuberculosis; UMD-CTIS: University of Maryland Global COVID-19 Trends and Impact Survey; UN IGME: United Nations Inter-agency Group for Child Mortality Estimation; UNAIDS: Joint United Nations Programme on HIV and AIDS; UNDP: United Nations Development Programme; UNICEF: United Nations Children’s Fund; WHO: World Health Organization.

a This is a count of unique health indicators and does not reflect duplicates caused by multiple disaggregation.

b Disaggregation for a given indicator, country and date depends on data availability.

c This is a count of the number of countries with data for at least one indicator and dimension of inequality within the dataset.

d SDG indicator data are published on the Global SDG Indicators Data Platform (https://unstats.un.org/sdgs/dataportal).

e Data are sourced from the WHO GHO portal (https://www.who.int/data/gho).

f Data for this dataset are available monthly from May 2020 to March 2022.

g Disaggregated estimates are published by the University of Maryland Social Data Science Center (https://covidmap.umd.edu/umdcsvs/Contingency_Tables/).

h Data for this dataset are available weekly since January 2020.

i Data come from disaggregated data published in the WHO COVID-19 Detailed Surveillance Data Dashboard (https://app.powerbi.com/view?r=eyJrIjoiYWRiZWVkNWUtNmM0Ni00MDAwLTljYWMtN2EwNTM3YjQzYmRmIiwidCI6ImY2MTBjMGI3LWJkMjQtNGIzOS04MTBiLTNkYzI4MGFmYjU5MCIsImMiOjh9).

j Data are sourced from the DHS Program Indicator Data API (https://dhsprogram.com/) and/or the UNICEF Data Warehouse (https://data.unicef.org/dv_index/).

k Data are sourced from the WHO Global Database on Child Growth and Malnutrition (https://platform.who.int/nutrition/malnutrition-database).

l Data are published by the UN IGME (https://childmortality.org/).

m Data are sourced from the Local and Small Area Estimation of IHME (https://ghdx.healthdata.org/local-and-small-area-estimation).

n Data are derived from UNAIDS/UNICEF/WHO annual estimates (https://aidsinfo.unaids.org).

o Data are sourced from the Eurostat data browser (https://ec.europa.eu/eurostat/databrowser).

p Data are sourced from the OECD data warehouse, OECD.Stat (https://stats.oecd.org/).

q Data are sourced from the WHO Global Health Estimates database (https://www.who.int/data/global-health-estimates).

r Data are sourced from the Global Burden of Disease Study 2019 (https://ghdx.healthdata.org/gbd-2019).

s Data are available from https://washdata.org/.

t Data are sourced from The World Bank Data Catalog (https://data.worldbank.org/).

u Data are sourced from the Global Data Lab website (https://globaldatalab.org/).

v Data are sourced from UNDP (https://hdr.undp.org/content/2021-global-multidimensional-poverty-index-mpi) or OPHI (https://ophi.org.uk/multidimensional-poverty-index/data-tables-do-files/).

## Data collected

The development of the WHO Health Inequality Data Repository involved an initial scoping exercise to identify relevant sources of disaggregated data. First, a preliminary list of data sources familiar to the development team was compiled, which included databases maintained by WHO as well as the United Nations and its specialized agencies (such as, the United Nations Statistics Division, United Nations Educational, Scientific and Cultural Organization, the United Nations Children’s Fund, the United Nations Development Programme, the Joint United Nations Programme on HIV/AIDS and the World Bank) and several other international health, development and research organizations (such as the United States Agency for International Development, the Institute for Health Metrics and Evaluation and the Organisation for Economic Co-operation and Development, among others). Additional searching and consultation were undertaken to identify other sources of data, focusing first on global datasets (containing data from any/all countries), and then on regional and continental datasets (containing data from multiple countries).

Next, the publicly available data for indicators within each candidate data source were assessed to determine relevance for inclusion in the Health Inequality Data Repository. The leading criterion was that the indicator data must be disaggregated by a dimension of inequality (a demographic, socioeconomic, geographical or other characteristic that is applied to define population subgroups within countries). We included only data reported at a country level; data pooled across multiple countries were excluded. Other strict inclusion criteria were that the dataset must be publicly available, accessible (either via an API or other data download functionality) and updated within the past 5 years (that is, since 2017).

Several other criteria were considered to guide the selection of data. In most cases, selected indicators pertained to health, though indicators related to the SDGs and determinants of health were also included (encompassing, for example, environmental health, women’s empowerment index, development indices and indicators, multidimensional poverty index and child protection indicators). Certain topics were prioritized because they were of special interest—for example, representing topics for which inequalities are understudied, such as indicators related to disability. Where available, age-standardized estimates tended to be prioritized over crude estimates. Indicators that expressed projections were not included. Duplicate or similar indicators were removed. Indicators measured as an absolute count or value (as opposed to a rate with a common scale) were excluded, as in the absence of a common indicator scale, data could not be compared across population subgroups and settings. (For example, an indicator of ‘total number of child deaths’ would be excluded, whereas an indicator of ‘child mortality rate per 1000 live births’ would be considered for inclusion.)

Further consideration was given to the quality of data sources, particularly the attributes of being timely, complete, accurate and reliable. To this end, we required that data include at least one estimate from 2015 or more recent to reflect the latest situation of inequality; if available, data from earlier time points were also included to enable comparisons of change over time. Indicators with a limited amount of disaggregated data, for example covering estimates from fewer than 10 countries, were not included. In cases where data sources included data from external sources, the original source was preferred (noting that there were exceptions for the SDG data, which were collected from the SDG Global Database, data from the World Bank and in cases where the extraction of data from the external original source was substantially less feasible). The availability of comprehensive indicator metadata was also a requirement for all data included in the Health Inequality Data Repository.

As a result of this initial scoping exercise, more than 50 potential data sources were considered and/or explored. The 2023 launch of the Health Inequality Data Repository included data from 16 sources. Relevant data from these sources were extracted, cleaned to remove data not meeting the inclusion criteria and to harmonize factors such as dimension of inequality and population subgroup names, and formatted according to the HEAT Plus Template specifications.^1^ As annual updates are undertaken, including the further exploration of country-level data sources, the data sources as well as the indicators featured in the Health Inequality Data Repository will be expanded.

## Data resource use

The Health Inequality Data Repository is intended for users with a range of technical skills, for the broad purposes of exploring, analysing and monitoring inequalities. The datasets of the Data Repository provide necessary data inputs to carry out the cycle of health inequality monitoring, an approach detailed in tools and resources developed by the WHO Health Inequality Monitoring team.^5^ To this end, the disaggregated data can be used to perform assessments of the state of inequality across featured health topics or settings, conduct ongoing monitoring and evaluation and serve as evidence to inform the development of policies and programmes.

To date, the data from previous versions of the Health Equity Monitor and the Health Inequality Data Repository have been featured in numerous published works, which address diverse health topics. For illustrative purposes, we will describe two specific use cases by the WHO Health Inequality Monitoring team; other WHO materials and resources are available through the Health Inequality Monitor website.^6^ First, data from datasets on the WHO Health Inequality Data Repository have been used to prepare global *State of Inequality* reports and accompanying interactive visuals and equity country profiles.^7–9^ These reports represent the first systematic global analysis of inequality in the featured topic or setting, assessing both the latest situation of inequality and changes over time. The 2015 *State of Inequality: Reproductive, Maternal, Newborn and Child Health* report used disaggregated data to exemplify the innovative application of electronic visualization technology for reporting health inequalities.^7^ The use of interactive technologies to convey inequality data has since been integrated across WHO health inequality monitoring outputs.^6^*State of Inequality: Childhood Immunization*, published in 2016, reported persistent economic- and education-related inequalities in many countries, despite national improvements in immunization coverage over the previous decade.^8^ These findings led to a series of further in-depth inequality analyses in the topic, such as an exploration of how vulnerability and advantage can compound across multiple dimensions of inequality within 10 priority countries.^10^ The reduction of unfair inequalities is a growing priority of global immunization initiatives: for example, the 2021–25 strategy adopted by Gavi, the Vaccine Alliance emphasizing equity as an organizing principle, ‘with a high ambition to reduce the number of under-immunised children and an intensified focus on reaching the unreached’.^11^ In 2021, *State of Inequality: HIV, Tuberculosis and Malaria* marked the first comprehensive global assessment of inequalities in the three diseases, including 32 health indicators and five dimensions of inequality.^9^ The report highlighted critical gaps in data availability and data quality where improvements to health information systems are needed to understand and address inequalities. The Global Fund to Fight AIDS, Tuberculosis and Malaria 2023–28 strategy highlights the importance of maximizing health equity, gender equality and human rights with an emphasis on strengthening the collection and use of high-quality disaggregated data to support data-driven decision-making.^12^

Second, the data in the Health Inequality Data Repository have been used to prepare comprehensive examples that serve as the basis for capacity-strengthening workshops and eLearning courses on health inequality monitoring. Enhanced training opportunities in health inequality monitoring are important to encourage the widespread use of disaggregated data and increase political will for the use of data to advance health equity. Capacity-strengthening workshops are delivered to groups of 20–30 people and are themed around a particular country/region or health topic. Participants are encouraged to prepare post-workshop inequality reports and promote regular inequality monitoring practices within their areas of work. For example, a series of workshops held in Indonesia led to the development of the *State of Health Inequality: Indonesia* report by collaborators from multiple institutions within the country.^13^^,^^14^ Health inequality monitoring eLearning courses offer a self-paced option for learning, available for free to global audiences.^15^ They demonstrate the application of disaggregated data to assess inequalities in topics such as: immunization; HIV, tuberculosis and malaria; and sexual, reproductive, maternal, newborn, child and adolescent health. Courses pertaining to the general steps of health inequality monitoring and the use of specific software programmes are also available.^16^

## Strengths and weaknesses

A major strength of the Health Inequality Data Repository is that it provides a single point of access to disaggregated data for health inequality monitoring, with a large bank of indicators and dimensions of inequality relevant to global initiatives. To our knowledge, it is the largest global data repository of disaggregated health data and provides access to disaggregated data for all SDG indicators, where these data are available. The data are available for exploration using HEAT, which further enhances their usability by diverse stakeholder groups. WHO resources are available to support capacity-building in health inequality monitoring, including an eLearning course dedicated to HEAT.^16^

WHO plans to update and maintain the Data Repository on a yearly basis, which will ensure that the data remain timely. Annual updates will also allow for the Data Repository to remain relevant to global health interests, as additional health topics and indicators can be added in response to emerging global initiatives and priorities.

The Health Inequality Data Repository is constrained by the health indicators and dimensions of inequality data that are available in the public domain. Therefore, it is not possible to ensure that all settings and population subgroups are represented across each topic. The lack of data pertaining to socioeconomic-related dimensions of inequality was a limiting factor for many health and health-related indicators. The different sources of data draw from diverse data collection and preparation protocols, which should be taken into account when interpreting the data and making comparisons of data across sources.

## Data resource access

The Health Inequality Data Repository is publicly available through the WHO Health Inequality Monitor [https://www.who.int/data/inequality-monitor/data]. The Data Repository landing page contains a comprehensive list of all datasets included in the repository, which is kept up to date and is available through the above link. Users can download datasets in spreadsheet format (as xlsx files) and can access the data via an OData API. Each dataset is accompanied by comprehensive indicator metadata, detailing: general information about the dataset; information about and links to the original data sources, including relevant quality or data availability considerations and criteria for inclusion (if applicable); and, for each indicator, specifications about the indicator definition, calculation and disaggregation, or links to the original indicator metadata where possible.

A key feature of the Health Inequality Data Repository is its compatibility with HEAT, the WHO flagship software for exploring and analysing disaggregated data.^1^ All datasets are integrated within HEAT, Built-in Database edition (available online), and are compatible with HEAT Plus, Upload Database edition. The toolkit is an interactive application to create customized views of data from any of the datasets in the repository, allowing users to select the health topic, indicators, settings and dimensions of inequality of interest. HEAT and HEAT Plus facilitates the exploration of disaggregated data and the calculation of summary measures of inequality, with options for benchmarking between multiple settings. Users can further customize the type of visual output for the selected data, choosing from different types of graphs, maps and tables, which can be downloaded and used in reporting.

Enquiries can be submitted to Katherine Kirkby [kirkbyk@who.int].

## Ethics approval

Ethics approval was not required as the WHO Health Inequality Data Repository contains data that are in the public domain.

## Data Availability

The data underlying this article are publicly available through the WHO Health Inequality Data Repository at [https://www.who.int/data/inequality-monitor/data].

## References

[1] World Health Organization. Health Equity Assessment Toolkit. *Health Inequality Monitor*. 2023. https://www.who.int/data/inequality-monitor/assessment_toolkit (10 May 2023, date last accessed).

[2] Kirkby K , Schlotheuber A, Vidal Fuertes C, Ross Z, Hosseinpoor AR. Health Equity Assessment Toolkit (HEAT and HEAT Plus): exploring inequalities in the COVID-19 pandemic era. Int J Equity Health 2022;21:172. 36471346 10.1186/s12939-022-01765-7 PMC9720922

[3] Hosseinpoor AR , Bergen N, Schlotheuber A, Victora C, Boerma T, Barros AJ. Data Resource Profile: WHO Health Equity Monitor (HEM). Int J Epidemiol 2016;45:1404–05e. 27694569 10.1093/ije/dyw176 PMC5100617

[4] World Health Organization. *Health Inequality Data Repository. Health Inequality Monitor*. 2023. https://www.who.int/data/inequality-monitor/health-inequality-data-repository (10 May 2023, date last accessed).

[5] Hosseinpoor AR , Bergen N, Kirkby K, Schlotheuber A. Strengthening and expanding health inequality monitoring for the advancement of health equity: a review of WHO resources and contributions. Int J Equity Health 2023;22:49. 36932363 10.1186/s12939-022-01811-4 PMC10022555

[6] World Health Organization. *Health Inequality Monitor*. 2023. https://www.who.int/data/inequality-monitor (10 May 2023, date last accessed).

[7] World Health Organization. State of Inequality: Reproductive, Maternal, Newborn and Child Health. Geneva: World Health Organization, 2015.

[8] World Health Organization. State of Inequality: Childhood Immunization. Geneva: World Health Organization, 2016.

[9] World Health Organization. State of Inequality: HIV, Tuberculosis and Malaria. Geneva: World Health Organization, 2021.

[10] World Health Organization. Explorations of Inequality: Childhood Immunization. Geneva: World Health Organization, 2018.

[11] Gavi, The Vaccine Alliance. *The Equity Goal (Phase 5).* 2022. https://www.gavi.org/our-alliance/strategy/phase-5-2021-2025/equity-goal (10 May 2023, date last accessed).

[12] Global Fund to Fight AIDS, Tuberculosis and Malaria. *Fighting Pandemics and Building a Healthier and More Equitable World: Global Fund Strategy (2023–2028).* 2021. https://www.theglobalfund.org/en/strategy/ (10 May 2023, date last accessed).

[13] Hosseinpoor AR , Nambiar D, Tawilah J et al Capacity building for health-inequality monitoring in Indonesia: enhancing the equity orientation of country health information system. Glob Health Action 2018;11:1419739. 29569528 10.1080/16549716.2017.1419739 PMC5912437

[14] World Health Organization. State of Health Inequality: Indonesia. Geneva: World Health Organization, 2017.

[15] Bergen N , Kirkby K, Baptista A et al Health inequality monitoring channel on OpenWHO: capacity strengthening through eLearning. Int J Equity Health 2022;21:133. 36100901 10.1186/s12939-022-01739-9 PMC9469832

[16] World Health Organization. *Health Inequality Monitoring.* OpenWHO. 2023. https://openwho.org/channels/inequality-monitoring (10 May 2023, date last accessed).

